# The Impact of a Multi-Pronged Intervention on Students’ Perceptions of School Lunch Quality and Convenience and Self-Reported Fruit and Vegetable Consumption

**DOI:** 10.3390/ijerph17165987

**Published:** 2020-08-18

**Authors:** Stephanie S. Machado, Lorrene D. Ritchie, Hannah R. Thompson, Kristine A. Madsen

**Affiliations:** 1 School of Public Health, University of California, Berkeley, CA 94704, USA; stephanie_machado@berkeley.edu (S.S.M.); thompsonh@berkeley.edu (H.R.T.); 2 Nutrition Policy Institute, University of California Agriculture and Natural Resources, Berkeley, CA 94704, USA; lritchie@ucanr.edu

**Keywords:** school lunch, nutrition, children, interventions, fruits and vegetables, perceptions

## Abstract

School lunch programs provide an opportunity to improve students’ diets. We sought to determine the impact of a multifaceted intervention (cafeteria redesigns, increased points-of-sale and teacher education) on secondary students’ perceptions of school-lunch quality and convenience and fruit and vegetable intake. Surveys (*n* = 12,827) from middle and high school students in 12 intervention and 11 control schools were analyzed. We investigated change in school-lunch perceptions and lunchtime and daily fruit and vegetable consumption from 2016 to 2018. Among 8th graders, perceptions that school lunch tastes good and that school lunch was enough to make students feel full increased 0.2 points (on a 5-point scale; *p* < 0.01) in intervention schools relative to control schools. Among 10th graders, lunchtime fruit and vegetable consumption increased 6% in intervention relative to control schools (*p* < 0.05 and *p* < 0.01 respectively). Daily fruit intake increased 0.1 cups/day in intervention relative to control schools among 9th graders (*p* < 0.01). This study provides important evidence on the limited effect of design approaches in the absence of meal changes. We observed only modest changes in school lunch perceptions and fruit and vegetable consumption that were not consistent across grades, suggesting that additional efforts are needed to improve school-lunch uptake.

## 1. Introduction

Less than one-quarter of adolescents in the United States meet national dietary recommendations for fruit or vegetable intake [1], and poor childhood dietary habits have been linked to adult chronic disease [2,3] and obesity [2,4]. As 20% of adolescents ages 12−19 are obese, dietary intake is critical to address [5]. The National School Lunch Program (NSLP), which has been shown to provide healthier meals than those brought from home or purchased elsewhere [6,7], could improve children’s dietary intake. However, while the NSLP has the potential to reach nearly every public-school student in the nation [8], participation rates are suboptimal. On a given day, only 52% of middle school students and 39% of high school students attending schools with the NSLP participate in the program [9]. Further, food waste in the NSLP remains high, especially for vegetables [10], while low vegetable consumption is a major contributor to chronic disease [2,3].

Perceptions of meal quality [11,12] and time available to eat during the lunch period [13,14] are two driving factors that influence school meal participation and consumption, respectively. The physical dining space [15] may impact meal perceptions. While interventions have incorporated minor cafeteria décor changes such as nutrition posters and student artwork [16], to our knowledge, no studies have explored expansive cafeteria décor and seating redesigns. Further, norms set by adults [17] may impact meal perceptions, yet it remains unknown if and/or how teachers influence youths’ perceptions of school meals. Finally, additional points of sale, such as vending machines and mobile carts, have potential to reduce school-lunch wait times, yet their efficacy in school-lunch programs remains unknown.

A multi-pronged intervention comprising cafeteria redesign, teacher education about school meals, and school meal sales through vending machines and mobile carts [18] was implemented in middle and high schools in an urban, low-income school district to improve NSLP participation and reduce food waste. A small relative increase in NSLP participation, the primary study outcome, was observed in the intervention schools compared to control schools [19] and food waste did not decrease in intervention schools [20]. In an effort to understand why the innovative intervention was not more impactful, we sought to explore the impact on the intervention’s proximal outcomes, student perceptions of meal quality and convenience, and student-reported fruit and vegetable consumption. Better understanding these outcomes can identify additional targets for intervention and enhance the likelihood that future interventions will achieve greater impacts.

## 2. Materials and Methods

A repeated cross-sectional, quasi-experimental design was used. Twenty-four schools (12 middle and 12 high schools) in a large, urban school district in California participated in the study from school years 2015−2016 (baseline) through 2017−2018. Twelve schools (average of 12,903 students per year in grades 6−12 across 3-year study period) received the intervention and 12 (average of 11,156 students per year in grades 6−12 across 3-year study period) served as control schools. Details on study design have been published previously [18]; briefly, 5 schools that had piloted the intervention prior to the study were assigned to the intervention and the remaining 19 schools were randomly assigned, stratifying on school type (middle vs. high) and an index of high vs. low need (based on student eligibility for free or reduced-price meals, percent who identify as White, and academic performance) [18].

The intervention comprised: (1) a cafeteria redesign to update décor, paint, and seating options in the cafeteria; (2) teacher education about school meals (newsletters and short videos) and school meal taste tests; and (3) complete school meals (including fruits and vegetables, per NSLP guidelines) sold through vending machines and mobile carts (students had the option of purchasing meals through the traditional serving line or via a mobile cart or vending machine). Mobile carts and vending machines were located in the cafeteria (*n* = 10 schools) or in the library (*n* = 1 school) or just outside the cafeteria (*n* =1 school). The intervention was rolled out over a 2-year period with full implementation by fall 2017. Control schools did not receive the intervention after the study period ended. This study uses data from student surveys administered in spring of 2016 (baseline) and 2018 (full intervention implementation). The study was approved by the Committee for Protection of Human Subjects at the University of California, Berkeley (protocol ID: 2014−12−7010; approval date 3/10/2015) and by the school district’s research office. Note that the research team does not have permission from the school district to make the student survey dataset publicly available.

### 2.1. Participants

All students in grades 7–10 in 2016 (*n* = 13,548) and grades 8−10 (*n* = 10,444) in 2018 were eligible to complete anonymous surveys, administered by teachers in homerooms in spring of 2016 and 2018. Surveys were also administered in spring 2017 but were not included in analyses as not all intervention schools had full implementation that school year [18]. Parent/guardian notices with opt-out slips were sent home with all students prior to student participation in the survey. Teachers were instructed to read students an assent script before students completed the survey. One control high school was dropped from the analysis because they did not complete surveys at baseline.

### 2.2. Intervention and Conceptual Framework

**Figure 1:**
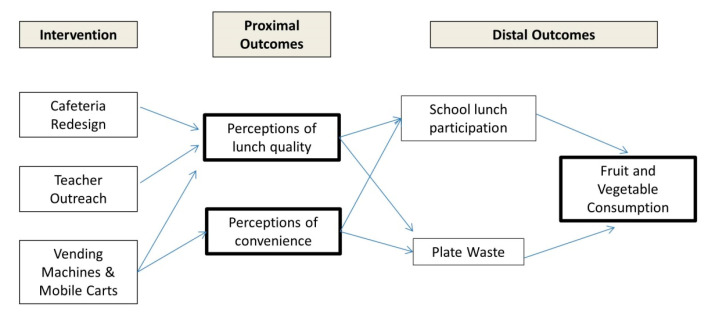
Conceptual Framework.

The intervention, which was grounded in principles of behavioral economics and social learning theory [18], included three components: cafeteria redesign, teacher outreach, and additional points of school lunch sale through mobile carts and vending machines. As depicted in Figure 1, the intervention was designed to improve student perceptions of school-lunch quality and convenience (proximal outcomes), which were expected to increase school-lunch participation, reduce food waste, and ultimately increase fruit and vegetable consumption by students (distal outcomes).

Proximal outcomes of the intervention included student perceptions of lunch quality and convenience. Definitions of school meal quality vary across studies. For example, quality is defined as taste, smell, texture, and preparation style by Zhao et al. [21] and as visual appeal by Gosliner [13]. Kwon et al. take a more comprehensive approach and define school meal quality as diversity of food and texture, taste, temperature, ingredient quality, nutritional quality, seasonality, appeal, and satiety [22]. Our study defines quality as taste, healthfulness, and satiety. Our proxy for convenience is lunch line length. While there is no standard definition for school meal convenience, ease of obtaining food [23,24] or quick access [24] are characteristics of convenience in the school nutrition literature.

The dining environment has been shown to predict middle school student satisfaction with the school meal program [25] and college student perceptions of meal acceptability [15]. Additional research shows that the same meal served in a school setting is perceived as less acceptable than when served in a restaurant setting [15]. Therefore, we posited that improving the school cafeteria environment with colorful paint and artwork and modern décor and furniture would improve student perceptions of school lunch. Adult modeling [26,27,28] and encouragement [27] of healthy eating have been associated with healthy dietary habits among children. Further, youth emphasized, in a qualitative study, that they would eat healthier if adults encouraged them to do so. [29] We therefore hypothesized that improvements in teacher modeling and encouragement of school lunch through teacher outreach would improve student perceptions of school lunch. Additionally, offering meals through visually appealing vending machines and mobile carts could affect perceptions of quality. Mobile carts and vending machines are also promising methods to improve the convenience of food delivery in a school setting [30,31]; we posited that delivering school lunch through vending machines and mobile carts would improve student perceptions of lunch line length.

Distal outcomes of the intervention included school-lunch participation (using district-provided participation data); school lunch waste (measured via aggregate weighing plate waste); and fruit and vegetable consumption at school and over the entire day (measured by the student survey). Perceptions of lunch quality are associated with school meal participation in prior studies. Smith et al. found taste, appeal, freshness, and healthfulness were among the top meal quality components middle school students list as reasons for non-participation [12]. Further, Marples et al. found that meal quality (general; no sub-items) was associated with high school student participation [11]. Therefore, we hypothesized that improvements in perceived lunch quality would lead to improvements in participation. Because students say they would be more likely to participate in school lunch if lines were shorter [12], we also hypothesized that improved perceptions of line length would increase school-lunch participation. As negative perceptions of NSLP lunch taste are barriers to reducing food waste, we posited that improvements in perceptions of school lunch quality would lead to a decrease in food waste [21].

Improvements in perceptions of line length, and thus more time available to eat, is posited to improve overall food waste and fruit and vegetable consumption. More time available to eat is associated with reductions in entrée, milk, and vegetable waste. [14] Gains in participation could also differentially improve fruit and vegetable consumption since NSLP lunches tend to have more fruits and vegetables than lunches brought from elsewhere, and students who participate in the NSLP consume more fruits and vegetables than those who do not [6,7].

### 2.3. Measures

Outcomes were based on student survey data. The variables of interest on the student survey included perceptions of school lunch, lunch-purchasing behavior, and lunchtime fruit and vegetable intake. Perception questions were adapted from the Healthy Eating, Active Communities Student Survey [32]. Students were asked if they agreed (5-point Likert scale from 1 = “Strongly Disagree” to 5 = “Strongly Agree”) with the statements: “the school lunch is enough to make me full”, “school lunch tastes good”, “school lunch is healthier than foods I bring from home or off-campus”, and “lunch lines are too long”. These variables were treated as continuous. Lunchtime intake of fruit (not including fruit juice), 100% fruit juice, vegetables (not including fried potatoes), and salad by students was assessed with the question “yesterday at lunch, how much did you eat” with response options “none”, “a little”, “some”, and “a lot”. For each of the 4 items, responses were collapsed into binary variables for none or any consumption. Fruit intake is reported as fruit (excluding juice), all fruit (fruit juice and/or fruit excluding juice), and vegetables (vegetables—excluding fried potatoes—and/or salad).

Fruit and vegetable consumption in a typical week was also measured to determine if the intervention influenced overall daily intake. Daily cup equivalents were estimated using the Block Kids Food Screener [33], which was selected given time and feasibility constraints in the school setting. The screener has been validated against a 24-h dietary recall, with correlations adjusted for within-subject variance of measures covering different time intervals of 0.53 and 0.60 for vegetables and fruit, respectively [33]. For each of the 12 types of fruits and vegetables listed, students were asked “how many days a week do you usually eat it?” (ordinal categorical variable, range 0–7) and “if you eat it, about how much in one day?” (ordinal categorical variable: “a little”, “some”, and “a lot”). NutritionQuest (Berkeley, CA, USA), the developer of the Block Screener, converted responses to daily cup equivalents of all fruits, fruits (excluding juice), all vegetables, and vegetables (excluding potatoes) consumed based on age and gender. Students who did not identify as male or female were excluded from the Block analysis as the analysis only assigns values to those with male or female gender. The complete survey, along with survey question sources can be found in the Supplement (Survey S1 and Survey Sources S2).

To allow for analyses restricted to students who eat the school lunch—as these are the students for whom we would expect the greatest change—students were also asked “where do you usually get your lunch on a school day?” and “did you eat school lunch yesterday?”. The first question was used to restrict the sample to those who typically eat the school lunch for school-lunch perception questions. The second question was used to restrict the sample to students who ate school lunch the day of the assessment for dietary intake questions.

### 2.4. Analysis

We conducted stratified analyses by grade, comparing baseline (*n* = 6502) to follow-up (*n* = 6325) responses in grades 8, 9, and 10 separately. To investigate change in school-lunch perceptions, we used linear random intercept models with restricted maximum-likelihood estimations to account for the small number of clusters. To investigate change in lunchtime fruit and vegetable consumption, we used logistic random intercept models. To determine change in daily fruit and vegetable consumption, we used generalized linear models (GLM) with a gamma family log link to allow us to account for multiple zeros and highly right skewed data on consumption. All models included a random effect for school to account for clustering by school and school-level (student enrollment and proportion of students eligible for free and reduced-price meals) and student-level (gender and race/ethnicity) covariates. All models investigated change between intervention and control schools from baseline to 2 years follow-up. All analyses were conducted in Stata version 15.1 (StataCorp, College Station, TX, USA).

## 3. Results

Among all eligible students in the 23 schools included in analysis, the mean response rates at baseline were 61% and 62% for intervention and control schools, respectively. At follow-up, the mean response rates were 62% and 56% for intervention and control schools, respectively. No significant differences were seen in response rates. The control school excluded from analysis due to missing data was slightly smaller, had a lower proportion of Asian students, and had a higher proportion of Latino/a students and students eligible for free or reduced price meals than the control schools included in the analysis. The final sample of students—comprising of those with complete demographic data in grades 8, 9 and 10 in 23 schools—includes 3551 from intervention schools and 2951 from control schools at baseline and 3257 and 3068 from intervention and control schools respectively at follow-up (*n* = 12,827 in total). The sample size varies slightly by survey question due to missing data. Table 1 presents demographic characteristics of the included survey respondents at baseline and follow-up.

At baseline among all students, 11% agreed or strongly agreed that school lunches were filling, 5% that school lunches tasted good, 10% that school lunches were healthy, and 47% that the lines were too long. Table 2 presents mean Likert-scale scores for school-lunch perception outcomes. There was a significant between-group difference in change in perceptions of taste and feeling full among 8th grade students only. Perceptions that school lunch tasted good (0.19: 95% CI: 0.07, 0.31, *p* < 0.01) and that lunch is enough to make them feel full (0.17: 95% CI: 0.04, 0.29, *p* < 0.01) significantly increased in intervention relative to control schools. No significant changes were seen in perceptions of the healthfulness of school lunch compared to other lunch options, or of line length, for any grade. When restricting the sample to students who typically ate school lunch, a relative increase in perceptions of fullness was seen for grade 10 only, (difference-in-change 0.37; 95% CI: 0.07, 0.67, *p* < 0.05) and no changes in perceptions of school lunch were seen in grades 8 and 9.

Table 3 and Table 4 present student-reported lunchtime and daily fruit and vegetable consumption, respectively. Significant relative increases in lunchtime consumption of all fruit (difference-in-change 6.2%; 95% CI: 1.0%, 11.5%, *p* < 0.05), and vegetables excluding fried potatoes (difference-in-change 6.1%; 95% CI: 1.5%, 10.7%, *p* < 0.01) were seen in grade 10 only. No changes were seen when restricting to those who ate school lunch the day of the assessment.

For weekly fruit and vegetable intake, a relative increase in fruit consumption was seen in grade 9 only (difference-in-difference 0.09 cups per day; 95% CI: 0.03, 0.15, *p* < 0.01). When restricting to those who ate school lunch the day of the assessment, relative decreases in all vegetables (difference-in-difference −0.32; 95% CI: −0.48, −0.16, *p* < 0.001) and vegetables excluding potatoes (difference-in-difference −0.29; 95% CI: −0.46, −0.12, *p* = 0.001) were seen for grade 10 students in intervention vs. control schools. No other changes were seen in the restricted sample.

## 4. Discussion

This study provides an important exploration of perceptions of school-lunch quality and convenience and fruit and vegetable intake. Results suggest that while the intervention was modestly effective in improving perceptions of lunch quality among students in grade 8, it was ineffective in improving perceptions of convenience among students in any grade, the hypothesized mechanisms of change. The results provide insight into why slight relative increases were seen in lunch participation [19] and student-reported fruit and vegetable consumption, while plate waste did not improve [20] over the course of the intervention.

Though perceptions of taste and feeling full marginally improved for 8^th^ grade students, students had poor perceptions of school lunch across all grades at baseline, with most students not agreeing that lunch was healthy or tasted good. Our findings on student-reported meal perceptions are lower than other studies exploring perceptions of meal quality during similar years. In a survey of middle school students, Smith et al. found 44% of students agreed that school meals tasted good and 65% agreed were healthy [12]. In another study on middle school student perceptions, Kjosen et al. found that the mean agreement score for school meal satisfaction (a composite of multiple quality attributes, including taste and appearance) was a 3, or “Agree a Little”, on a 4-Point Likert scale [34]. Other studies also indicate that students want higher-quality food at school [29,35]. Our study suggests that simply changing how meals are served is insufficient to improve perceptions; changes in the quality of school meals may be required. In our study, no changes were made to the meals.

The intervention did not appear to have an effect on perceptions of school-lunch convenience as perceptions of lunch lines remained unchanged in both intervention and control schools. Further, student agreement that lunch lines were too long remained high despite the additions of a vending machine and mobile cart at each intervention school. As we documented previously, all three potential points of lunch sale (regular lunch lines, vending machines, and mobile carts) were only in operation together 27% of the days during the intervention period. On days when both vending machines and mobile carts were operating in intervention schools, only 4% of meals were sold from vending machines and 12% were sold from mobile carts [20]. The vast majority of students still used the regular lunch line. Thus, the vending machines and mobile carts did not appear to do as much as expected to expand the number of points of access to school lunch as anticipated, and was not sufficient to alter perceptions of line length. Qualitative interviews with staff implementing the intervention suggest that the operational inconsistency of the mobile carts and vending machines, paired with regulatory constraints that limited location options, may have contributed to low use of the additional points of sale [36]. Future studies are needed to explore how to market and operate additional points of sale to improve student uptake of school meals.

The intervention appeared to improve lunchtime fruit and vegetable consumption for 10th grade students only. However, the association disappeared for fruits and vegetables when restricting to those who ate school lunch the day of the assessment, the very students among whom we would expect to see an increase. Further, in this restricted sample for grade 10, the intervention group saw decreases in daily consumption of vegetables relative to the control group. In addition, our objective plate waste data from this study show relative increases in fruit and vegetable waste in intervention schools versus control schools [20]. These discrepancies suggest that the intervention does not increase fruit and vegetable intake. Qualitative studies suggest that students want better tasting school meals [29,35]. Asada found that the lack of visual appeal of the fruits and vegetables served in school meals is a primary reason why students do not eat them [35]. There is some evidence that the visual appeal of fruit in school lunch is positively associated with fruit intake [13]. Future interventions might address fruit and vegetable appeal as a method to improve perceptions and consumption.

Findings on perceptions of lunch quality and convenience add to our understanding of why school lunch participation, plate waste, and fruit and vegetable consumption (our distal outcomes) did not markedly improve. It is possible that though some meal quality indicators improved slightly, the inconsistencies across grades and the small magnitude of change did not translate into improvements in overall school meal participation or plate waste. Further, students still did not have enough time to eat, as evidenced by the lack of improvement in perceptions of lunch line length, and thus plate waste and fruit and vegetable consumption did not improve.

This study has several limitations. As only anonymous data were collected, we were unable to link individual student observations at baseline and follow-up, limiting our ability to include all students in the analyses and utilize a longitudinal design. Further, the full intervention was only in place for one school year instead of two, as originally planned. The delayed roll-out of the intervention, accompanied by the inconsistent operation of the vending machines and mobile carts, reduced the intended intervention dose and may have contributed to null findings. As lunchtime fruit and vegetable intake was assessed with only a semi-quantitative measure, and as the Block Screener demonstrates moderate validity [33] for daily fruit and vegetable consumption measurement, the data may not reflect objective consumption. Interpretations of lunch quality and convenience vary across studies, and our definitions do not align with all other studies in the field. There is a need for validation studies of school meal quality and convenience constructs. Finally, due to the limited determined impact on the distal outcomes [19,20] we were unable to conduct a mediation analysis to assess the validity of our conceptual model. Future studies should formally test similar conceptual models for improving lunch participation and reducing plate waste.

## 5. Conclusions

We hypothesized that an intervention involving cafeteria redesigns, teacher outreach, and mobile carts and vending machines would increase student perceptions of lunch quality and convenience by middle and high school students, which in turn, would result in improved student participation and intake of school lunch. We observed only modest changes in perceptions of school meals that were not consistent across grades, suggesting that additional efforts are needed to impact changes in student uptake of school meals. Given that virtually all children in the U.S. have access to school meals and that school meals are healthier than what students typically consume elsewhere, it is critical to identify additional ways to improve school meal uptake by students.

## Figures and Tables

**Table 1:** Demographic characteristics of survey sample.

Sample Characteristics	Surveys from Intervention Schools ^a,b^	Surveys from Control Schools ^a,b^	*p*-Value ^c^
**Race/Ethnicity, *n* (%)**			<0.001
African American	264 (4%)	222 (4%)	
Asian	3548 (52%)	2822 (47%)	
Latino	1680 (25%)	1,130 (19%)	
White	426 (6%)	926 (15%)	
Other ^e^	890 (13%)	919 (15%)	
**Gender, *n* (%)**			<0.001
Female	3153 (46%)	3206 (53%)	
Male	3525 (52%)	2664 (44%)	
Other ^f^	130 (2%)	149 (2%)	
**Grade 8, *n* (%)**	1969 (29%)	2085 (35%)	<0.001 ^d^
Ate school lunch yesterday	456 (24%)	456 (22%)	0.377
Typically eat school lunch	413 (22%)	429 (22%)	0.808
**Grade 9, *n* (%)**	2534 (37%)	1883 (31%)	<0.001 ^d^
Ate school lunch yesterday	615 (25%)	267 (14%)	<0.001
Typically eat school lunch	572 (24%)	260 (15%)	<0.001
**Grade 10, *n* (%)**	2305 (34%)	2051 (34%)	<0.001 ^d^
Ate school lunch yesterday	602 (27%)	390 (19%)	<0.001
Typically eat school lunch	572 (26%)	358 (18%)	<0.001

^a^ N’s include respondents with complete race/ethnicity, grade, and gender data. ^b^ While stratified (within-grade) analyses compared unique groups of students, some students are counted in both grades 8 and 10, reflecting the longitudinal nature of the study. Surveys are anonymous. ^c^ P-values based on Chi^2^ tests. ^d^ *P*-value for differences in proportion in grades 8, 9 and 10 for intervention vs. control schools. ^e^ Includes respondents who identify as multiple or other races/ethnicities or declined to state their race/ethnicity. ^f^ Includes respondents who identify as other gender or declined to state their gender.

**Table 2:** Student-reported school lunch perceptions, to follow-up ^a^.

	Intervention Schools			Control Schools			Between-Group Difference in Change ^b^ 95% CI
Grade	Baseline (*n* = 3551) Mean ^c^ ± SE	Follow-Up (*n* = 3257) Mean ± SE	Difference Mean 95% CI	Baseline (*n* = 2951) Mean ± SE	Follow-Up (*n* = 3068) Mean ± SE	Difference Mean 95% CI	
School lunch is enough to feel full							
grade 8	2.39 ± 0.05	2.43 ± 0.05	0.04 −0.05, 0.14	2.44 ± 0.04	2.32 ± 0.05	−0.12 ** −0.21, −0.04	0.17 ** 0.04, 0.29
grade 9	2.55 ± 0.06	2.61 ± 0.06	0.06 −0.01, 0.14	2.41 ± 0.07	2.43 ± 0.07	0.02 −0.06, 0.11	0.04 −0.08, 0.16
grade 10	2.51 ± 0.04	2.55 ± 0.04	0.04 −0.04, 0.12	2.47 ± 0.04	2.39 ± 0.04	−0.08 −0.16, 0.01	0.12 0.00, 0.23
School lunch tastes good							
grade 8	2.01 ± 0.06	2.01 ± 0.06	0.00 −0.08, 0.09	2.08 ± 0.05	1.89 ± 0.06	−0.18 *** −0.27, −0.10	0.19 ** 0.07, 0.31
grade 9	2.31 ± 0.07	2.34 ± 0.07	0.02 −0.05, 0.10	2.04 ± 0.08	2.09 ± 0.08	0.05 −0.04, 0.13	−0.02 −0.14, 0.09
grade 10	2.34 ± 0.06	2.34 ± 0.06	0.00 −0.08, 0.08	2.18 ± 0.07	2.20 ± 0.07	0.02 −0.07, 0.10	−0.02 −0.13, 0.10
School lunch is healthier than foods I bring from home or off−campus							
grade 8	2.28 ± 0.04	2.25 ± 0.04	−0.03 −0.12, 0.06	2.29 ± 0.04	2.17 ± 0.04	−0.12 ** −0.21, −0.03	0.09 −0.03, 0.22
grade 9	2.49 ± 0.04	2.51 ± 0.04	0.03 −0.05, 0.10	2.31 ± 0.05	2.38 ± 0.05	0.07 −0.02, 0.16	−0.05 −0.17, 0.07
grade 10	2.50 ± 0.04	2.53 ± 0.04	0.03 −0.05, 0.11	2.33 ± 0.05	2.38 ± 0.05	0.05 −0.04, 0.13	−0.02 −0.13, 0.10
Lines are too long							
grade 8	3.62 ± 0.10	3.67 ± 0.10	0.05 −0.04, 0.14	3.60 ± 0.09	3.60 ± 0.09	0.00 −0.08, 0.09	0.05 −0.08, 0.17
grade 9	3.49 ± 0.15	3.39 ± 0.15	−0.10 * −0.17, −0.02	3.28 ± 0.18	3.21 ± 0.18	−0.08 −0.16, 0.01	−0.02 −0.14, 0.09
grade 10	3.55 ± 0.18	3.49 ± 0.18	−0.06 −0.14, 0.01	3.25 ± 0.21	3.12 ± 0.21	−0.13 ** −0.21, −0.05	0.07 −0.05, 0.18

Values based on linear random intercept models with school as a random effect and adjusting for school-level enrollment and percent free and reduced price meal eligibility and student-level gender and race and ethnicity. ^a^ N’s include respondents with complete race/ethnicity, grade, and gender data. N’s vary slightly by item due to missingness. Missingness range 1–2%. Baseline: *n* = 2088 in grade 8, 2237 in grade 9, 2177 in grade 10; Follow-Up: *n* = 1966 in grade 8, 2180 in grade 9, 2179 in grade 10. ^b^ Difference in change from baseline to follow-up in intervention schools compared to control schools. ^c^ 5-point Likert scale from 1–5 (“Strongly Disagree” to “Strongly Agree”) * *p* < 0.05 ** *p* ≤ 0.01 *** *p* = 0.01.

**Table 3:** Estimated-percentage of students consuming fruits and vegetables “yesterday”, baseline to follow-up ^a^.

	Intervention Schools			Control Schools			Between-Group Difference in Change
Grade	Baseline (*n* = 3551) Mean ± SE	Follow-Up (*n* = 3257) Mean ± SE	Difference Mean 95% CI	Baseline (*n* = 2951) Mean ± SE	Follow-Up (*n* = 3068) Mean ± SE	Difference Mean 95% CI	
Ate fruit at lunch yesterday (excluding fruit juice), % Agree							
grade 8	56.9 ± 3.5%	56.6% ± 3.8%	−0.3% −7.1%, 6.4%	61.4% ± 3.0%	56.4% ± 3.0%	−4.9% −10.6%, 0.8%	4.6% −4.4%, 13.6%
grade 9	56.7% ± 2.8%	52.8% ± 3.4%	−3.9% −10.0%, 2.1%	57.3% ± 2.7%	54.7% ± 3.1%	−2.7% * −5.0%, −0.3%	−1.3% −7.7%, 5.2%
grade 10	60.5% ± 3.2%	61.3% ± 3.2%	0.8% −3.7%, 5.3%	56.5% ± 3.3%	52.5% ± 4.4%	−4.0% * −7.8%, −0.3%	4.8% −1.0%, 10.6%
Ate fruit at lunch yesterday (including fruit juice), % Agree							
grade 8	59.9% ± 3.8%	59.1% ± 3.6%	−0.8% −7.4%, 5.8%	65.1% ± 2.6%	58.3% ± 2.8%	−6.8% * −12.8%, −0.7%	6.0% −3.2%, 15.2%
grade 9	60.2% ± 2.4%	57.0% ± 2.9%	−3.2% −8.0%, 1.7%	61.6% ± 2.6%	57.0% ± 2.7%	−4.7% *** −7.5%, −1.9%	1.5% −4.0%, 7.0%
grade 10	62.3% ± 2.8%	63.3 ± 2.9%	1.0% −3.7%, 5.6%	61.8% ± 3.1%	56.6% ± 4.0%	−5.3% *** −8.0%, 2.5%	6.2% * 1.0%, 11.5%
Ate vegetables at lunch yesterday (excluding fried potatoes), % Agree							
grade 8	40.9% ± 3.6%	45.6% ± 3.9%	4.7% −0.3%, 9.7%	47.5% 3.8%	46.1% 3.4%	−1.4% −5.6%, 2.8%	6.1% −0.5%, 12.6%
grade 9	47.1% ± 3.2%	49.3% ± 2.1%	2.3% −4.0%, 8.5%	49.5% ± 3.2%	49.2% ± 2.4%	−0.3% −7.3%, 6.7%	2.5% −6.8%, 11.9%
grade 10	53.1 ± 2.1%	56.0% ± 2.4%	2.9% −0.9%, 6.6%	50.6% ± 2.0%	47.3% ± 1.8%	−3.3% * −6.3%, 0.2%	6.1% ** 1.5%, 10.7%

Values (obtained using Stata’s post estimation *margins* command) are marginal estimates from based on logistic random intercept models with school as a random effect and adjusting for school-level free or reduced price meal eligibility and enrollment and student-level gender and race/ethnicity. ^a^ N’s include respondents with complete race/ethnicity, grade, and gender data. N’s vary slightly by item due to missingness. Missingness range 1–3%. 2016: *n* = 2088 in grade 8, 2237 in grade 9, 2177 in grade 10); 2018; *n* = 1966 in grade 8, 2180 in grade 9, 2179 in grade 10. * *p* < 0.05 ** *p* < 0.01 *** *p* ≤ 0.001.

**Table 4:** Estimated mean weekly fruit and vegetable consumption (cups/day), baseline to follow-up ^a^.

	Intervention Schools			Control Schools			Between-Group Difference in Change ^b^ 95% CI
Grade	Baseline (*n* = 3487) Mean ± SE	Follow-Up (*n* = 3187) Mean ± SE	Difference Mean 95% CI	Baseline (*n* = 2885) Mean ± SE	Follow-Up (*n* = 2978) Mean ± SE	Difference Mean 95% CI	
Fruit (excluding fruit juice), Cups/Day							
grade 8	1.08 ± 0.03	1.11 ± 0.04	0.03 −0.09, 0.15	1.00 ± 0.02	0.97 ± 0.04	−0.03 −0.10, 0.04	0.06 −0.08, 0.19
grade 9	0.96 ± 0.05	0.90 ± 0.04	−0.06 −0.12, 0.00	1.07 ± 0.05	0.94 ± 0.04	−0.13 *** −0.18, −0.08	0.07 −0.01, 0.14
grade 10	0.88 ± 0.03	0.89 ± 0.04	0.00 −0.10, 0.11	0.98 ± 0.03	0.98 ± 0.02	0.00 −0.07, 0.08	0.00 −0.14, 0.15
All fruit, Cups/Day							
grade 8	1.39 ± 0.04	1.42 ± 0.06	0.04 −0.12, 0.19	1.36 ± 0.03	1.30 ± 0.04	−0.06 −0.13, 0.02	0.07 −0.06, 0.20
grade 9	1.25 ± 0.05	1.17 ± 0.03	−0.08 ** −0.13, −0.03	1.39 ± 0.05	1.18 ± 0.03	−0.21 *** −0.27, −0.15	0.09 ** 0.03, 0.15
grade 10	1.17 ± 0.03	1.17 ± 0.04	0.00 −0.09 0.09	1.29 ± 0.04	1.24 ± 0.03	−0.05 −0.14, 0.03	0.04 −0.06, 0.14
Vegetables (excluding potatoes), Cups/Day							
grade 8	0.70 ± 0.02	0.74 ± 0.04	0.04 −0.02, 0.10	0.66 ± 0.03	0.68 ± 0.03	0.02 −0.04, 0.07	0.02 −0.09, 0.14
grade 9	0.66 ± 0.03	0.65 ± 0.03	−0.01 −0.05, 0.03	0.70 ± 0.04	0.67 ± 0.03	−0.03 −0.12, 0.05	0.03 −0.11, 0.18
grade 10	0.73 ± 0.04	0.77 ± 0.03	0.04 −0.06, 0.14	0.72 ± 0.02	0.79 ± 0.03	0.07 * 0.01, 0.13	−0.04 −0.19, 0.11
All vegetables, Cups/Day							
grade 8	0.99 ± 0.02	1.0 ± 0.04	0.01 −0.07, 0.09	0.93 ± 0.04	0.95 ± 0.04	0.02 −0.08, 0.12	−0.01 −0.14, 0.12
grade 9	0.90 ± 0.03	0.88 ± 0.03	−0.02 −0.07, 0.02	0.97 ± 0.06	0.94 ± 0.04	−0.04 −0.17, 0.10	0.02 −0.14, 0.17
grade 10	0.95 ± 0.04	0.99 ± 0.03	0.04 −0.07, 0.16	0.97 ± 0.03	1.05 ± 0.04	0.07 −0.01, 0.15	−0.03 −0.16, 0.11

Values (obtained using Stata’s post estimation *margins* command) are marginal estimates from generalized linear models with a Gamma family log link, clustering by school, and adjusting for school-level free or reduced price meal eligibility and enrollment and student-level gender and race/ethnicity. ^a^ N’s include respondents with complete race/ethnicity and grade data and those identifying as male or female gender. N’s vary slightly by item due to missingness. Missingness < 1%. Baseline: *n* = 2040 in grade 8, 2192 in grade 9, 2140 in grade 10); Follow-Up: *n* = 1904 in grade 8, 2129 in grade 9, 2132 in grade 10. ^b^ Relative percent change from baseline to follow-up in mean cups/day in intervention schools compared to control schools. * *p* < 0.05 ** *p* < 0.01 *** *p* ≤ 0.001.

## Supplementary Materials

The following are available online at https://www.mdpi.com/1660-4601/17/16/5987/s1, Survey S1. Student Survey. Survey Sources S2. Student Survey Sources.
