# Supplementary material for: The Impact of a Multi-Pronged Intervention on Students’ Perceptions of School Lunch Quality and Convenience and Self-Reported Fruit and Vegetable Consumption
Source: Int J Environ Res Public Health. 2020 Aug 18;17(16):5987. doi: 10.3390/ijerph17165987 (PMC7460536; doi:10.3390/ijerph17165987)
Supplement: Supplementary file 1 [file ijerph-17-05987-s001.zip › Student Survey.pdf]

SFUSD School Nutrition Survey

School Name: ________________________________ **Today's Date:** ____/____/____

This survey is about what students eat. Your answers will help improve your school's lunch program. You do not have to complete the survey, but we hope that you will. This is a chance for you to have your voice heard.

The survey is anonymous, so no one will know what you answered. Please do not write your name on the survey. This survey is being conducted by SFUSD and the University of California.

Remember:

This is not a test! There are no right or wrong answers. We want to know about you and what you think.

Read each question carefully. Think about it before you choose an answer.

Fill in each circle completely. If you want to change an answer, erase it completely before marking another answer.

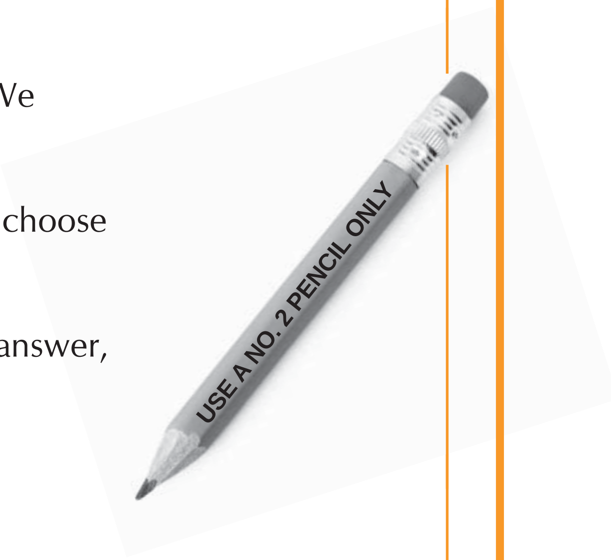

Incorrect Marks ✔ ✘ ⬬ • **Correct Mark** ●

1. Yesterday at lunch, how much of the following items did you eat or drink?

	None	A little	Some	A lot
a. **French fries** or other fried potatoes	○	○	○	○
b. **Green salad**	○	○	○	○
c. **Vegetables** (Do **not** include green salad, French fries or other fried potatoes)	○	○	○	○
d. **Fruit** (Do **not** include fruit juice)	○	○	○	○
e. **100% real fruit juices, like orange juice, apple juice or grape juice** (Do **not** include fruit-flavored drinks, Sunny Delight, aguas frescas, sport drinks, sweetened tea, Vitamin Water, Kool-Aid, etc.)	○	○	○	○

2. Did you eat the school breakfast yesterday?

○ Yes ○ No

3. Did you eat the school lunch yesterday?

○ Yes ○ No

4. Where do you usually get your lunch on a school day? *(Mark only one answer)*

- ○ I eat food from home
- ○ I eat food from the cafeteria or beanery
- ○ I eat food from a vending machine at school
- ○ I eat food from a food cart at school
- ○ I eat food from a friend's lunch
- ○ I buy or order food from off campus
- ○ I usually don't eat lunch

5. How much do you *agree or disagree* with the following statements about the *school lunch*?

	Strongly disagree	Disagree	Neutral	Agree	Strongly agree
a. The school lunch tastes good.	○	○	○	○	○
b. My friends eat the school lunch.	○	○	○	○	○
c. The lunch lines are too long.	○	○	○	○	○
d. I like food that I can buy off campus or bring from home better.	○	○	○	○	○
e. I like the fruits served with school lunch.	○	○	○	○	○
f. I like the vegetables served with school lunch.	○	○	○	○	○
g. The school lunch is enough food to make me full.	○	○	○	○	○
h. I get tired of the same foods being served for the school lunch.	○	○	○	○	○
i. Adults at school encourage me to eat the school lunch.	○	○	○	○	○
j. The school lunch is healthier than foods I bring from home or can buy off campus.	○	○	○	○	○

6. Mark all the places that you got your lunch on school days last week *(you can mark more than one answer)*:

- ◯ I ate food from home
- ◯ I ate food from the cafeteria or beanery
- ◯ I ate food from a vending machine at school
- ◯ I ate food from a food cart at school
- ◯ I ate food from a friend's lunch
- ◯ I bought or ordered food from off campus
- ◯ I didn't eat lunch

Think about EVERYTHING you drink in a typical week, including what you have at breakfast, lunch, dinner, after school, while watching TV, at bedtime, and on the weekend.

	**7. How many days a week do you usually drink it?**						**8. If you drink it, about how many times per day?**		
	**None**	**1 day**	**2 days**	**3-4 days**	**5-6 days**	**Every day**	**1**	**2**	**3 +**
a. Chocolate or other flavored milk	◯	◯	◯	◯	◯	◯	◯	◯	◯
b. Plain milk (Not chocolate or flavored; do **not** count milk on cereal)	◯	◯	◯	◯	◯	◯	◯	◯	◯
c. Sodas like Coke, Sprite, Sunkist, or grape soda (Do **not** include diet)	◯	◯	◯	◯	◯	◯	◯	◯	◯
d. Diet sodas like Diet Coke, Diet Dr. Pepper, Diet 7-Up, or Diet Sprite	◯	◯	◯	◯	◯	◯	◯	◯	◯
e. Sports drinks like Gatorade, Powerade, or Vitamin Water (Do **not** include diet)	◯	◯	◯	◯	◯	◯	◯	◯	◯
f. Energy drinks like Red Bull or Monster (Do **not** include diet)	◯	◯	◯	◯	◯	◯	◯	◯	◯
g. Sweetened coffees like mochas and Frappuccinos	◯	◯	◯	◯	◯	◯	◯	◯	◯
h. Sweetened teas like Snapple or Arizona	◯	◯	◯	◯	◯	◯	◯	◯	◯
i. Sweetened drinks with boba	◯	◯	◯	◯	◯	◯	◯	◯	◯
j. Unsweetened water (Tap, bottled, or carbonated)	◯	◯	◯	◯	◯	◯	◯	◯	◯
k. Drinks like punch, Kool-Aid, Tampico, Lemonade, or Sunny D (Do **not** include 100% juice)	◯	◯	◯	◯	◯	◯	◯	◯	◯
l. 100% real fruit juices like orange juice, apple juice, or grape juice	◯	◯	◯	◯	◯	◯	◯	◯	◯

Think about EVERYTHING you eat in a typical week, including what you have at breakfast, lunch, dinner, after school, while watching TV, at bedtime, and on the weekend.

9. How many days a week do you usually eat it? / **10. If you eat it, about how much in one day?**

	9. None	9. 1 day	9. 2 days	9. 3-4 days	9. 5-6 days	9. Every day	10. A little	10. Some	10. A lot
a. Apples, bananas, or oranges	○	○	○	○	○	○	○	○	○
b. Applesauce, fruit cocktail	○	○	○	○	○	○	○	○	○
c. Any other fruit, like strawberries or grapes	○	○	○	○	○	○	○	○	○
d. French fries, hash browns, tater tots	○	○	○	○	○	○	○	○	○
e. Other potatoes, like mashed or boiled	○	○	○	○	○	○	○	○	○
f. Ketchup or salsa	○	○	○	○	○	○	○	○	○
g. Green salad	○	○	○	○	○	○	○	○	○
h. Tomatoes, including on salad	○	○	○	○	○	○	○	○	○
i. Green beans or peas	○	○	○	○	○	○	○	○	○
j. Other vegetables like corn, carrots, broccoli	○	○	○	○	○	○	○	○	○
k. Vegetable soup, tomato soup, any soup or stew with vegetables in it	○	○	○	○	○	○	○	○	○

Information about You

11. How do you describe yourself? *(You can mark more than one answer if that is how you describe yourself)*

- ○ Asian
- ○ American Indian or Alaska Native
- ○ Hawaiian or Pacific Islander
- ○ Black or African American
- ○ Hispanic or Latino/Latina
- ○ White or Caucasian
- ○ Other ______________________

12. What grade are you in?

- ○ 7th grade
- ○ 8th grade
- ○ 9th grade
- ○ 10th grade
- ○ Other

13. Are you...

- ○ Female (Girl)
- ○ Male (Boy)
- ○ Other

14. How far in school did your mother go? (Indicate the highest level)

- ○ Did not finish high school
- ○ Finished high school or got GED
- ○ Did some college or training after high school
- ○ Finished college
- ○ I don't know

Thank you!
