# Supplementary material for: The Impact of a Multi-Pronged Intervention on Students’ Perceptions of School Lunch Quality and Convenience and Self-Reported Fruit and Vegetable Consumption
Source: Int J Environ Res Public Health. 2020 Aug 18;17(16):5987. doi: 10.3390/ijerph17165987 (PMC7460536; doi:10.3390/ijerph17165987)
Supplement: Supplementary file 1 [file ijerph-17-05987-s001.zip › Survey Question Adaptations.docx]

S2. Student Survey Sources

| **Survey Question** | **Adapted From** |
| --- | --- |
| The school lunch is enough to make me full | HEAC^a^ |
| School lunch tastes good | HEAC |
| School lunch is healthier than foods I bring from home or off-campus | Original^b^ |
| Lunch lines are too long | Original |
| Yesterday at lunch, how much did you eat: french fries or other fried potatoes | HEAC |
| Yesterday at lunch, how much did you eat: green salad | HEAC |
| Yesterday at lunch, how much did you eat: fruit (do not count fruit juice) | HEAC |
| Yesterday at lunch, how much did you drink: 100% fruit juice | HEAC |
| How many days a week do you usually eat/drink [fruit or vegetable]^d^: | BKFS^c^ |
| If you eat/drink [fruit or vegetable]^d^, about how much in one day? | BKFS^c^ |

^a^Healthy Eating, Active Communities Student Nutrition and Physical Activity Survey. ^b^Original survey question developed by the study team. ^c^Block Kids Food Screener. ^d^ (1) 100% real fruit juices, like orange juice, apple juice, or grape juice; (2) apples, bananas, or oranges; (3) applesauce, fruit cocktail; (4) any other fruit, like strawberries or grapes; (5) french fries, hash browns, tater tots; (6) other potatoes, like mashed or boiled; (7) ketchup or salsa; (8) green salad; (9) tomatoes, including on salad; (10) green beans or peas; (11) other vegetables, like corn, carrots, broccoli; (12) vegetable soup, tomato soup, any soup or stew with vegetables in it
